# Effect of omega-3 supplementation on metabolic and inflammatory markers in adults with HIV infection: a systematic review and meta-analysis

**DOI:** 10.3389/fnut.2026.1746723

**Published:** 2026-03-10

**Authors:** Jie Bai, Yuanxing Cao, Lichao Wang, Jiankang Ni, Yuyuan Zhu, Tong Wei

**Affiliations:** College of Medicine, Lishui University, Lishui, China

**Keywords:** human immunodeficiency virus, medical virology, meta-analysis, nutrition, omega-3

## Abstract

**Background:**

People living with human immunodeficiency virus (HIV) infection frequently exhibit altered lipid profiles and persistent inflammation that contribute to long-term morbidity. Omega-3 fatty acids are commonly recommended in this population, but the magnitude and consistency of their benefits remain uncertain. This study aimed to precisely re-estimate the effects of omega-3 supplementation compared with control on selected metabolic (lipid profile) and inflammatory biomarkers (C-reactive protein, CRP), IL-6, and TNF-*α* in adults with HIV.

**Methods:**

We searched PubMed/MEDLINE, Web of Science, and Scopus databases from 1965 to September 2025 for randomized trials reporting lipid or inflammatory biomarkers in adults with HIV. Eligible studies included participants aged 18 years or older and provided the exact numeric triplets required for reproduction (N, mean change or endpoint, and SD). Risk of bias was assessed using RoB 2. All analyses, figures, funnel plots, Egger and Begg tests, and subgroup tests were reproduced exactly from the investigator-supplied Stata 17.0. To explore heterogeneity, we conducted subgroup analyses and meta-regression analyses using study-level percentages of male participants and follow-up duration in months extracted from the data sheets; outcomes with fewer than 3 usable studies were not pooled.

**Results:**

Twenty-one trials supplying the exact numeric triplets met the inclusion criteria, representing 1,118 participants in total. The reproduced pooled analyses included total cholesterol (*n* = 15), High-Density Lipoprotein Cholestrol (HDL-C) (*n* = 13), triglycerides (*n* = 13), Low-Density (LDL-C) (*n* = 11), Interleukin-6 Lipoprotein Cholestrol (IL-6) (*n* = 4), CRP (*n* = 8), apolipoprotein (Apo A) (*n* = 4), and Apo B (*n* = 4). Using only the numeric outcome values explicitly reported in the included studies, we observed a modest, non-significant increase in HDL-C (weighted mean difference [WMD] 0.02 mg/dL, 95% CI –1.01–0.06) and a clear reduction in triglycerides (WMD − 0.86 mg/dL, 95% CI −1.18 to −0.54), while findings for total cholesterol and LDL-C were inconsistent and imprecise.

**Conclusion:**

In adults with HIV, omega-3 supplementation was associated with small, insignificant increases in HDL-C and meaningful reductions in triglycerides, whereas effects on other lipid fractions were inconsistent. Omega-3 supplementation was associated with a consistent reduction in CRP and modest improvements in other inflammatory biomarkers such as IL-6, while evidence for TNF-*α* remains inconclusive.

## 1 Introduction

The metabolic and inflammatory dysregulation in patients suffering from human immunodeficiency virus (HIV) infection constitutes the major comorbidities. With a global expansion of antiretroviral therapy, the management of HIV infection has experienced a paradigm shift, with a greater focus on long-term complications derived from either HIV-related pathomechanism or anti-retroviral therapy (ART)-induced side effects (1). ART regimens, particularly those containing protease inhibitors and certain integrase inhibitors, contribute to significant disturbances in lipid homeostasis and systemic inflammation, thereby elevating cardiovascular risk even with a suppressed viremia (2–4). In addition, a chronic immune activation in HIV infection, marked by elevated C-reactive protein (CRP), interleukin-6 (IL-6), and tumor necrosis factor-alpha (TNF-*α*), has been consistently observed and is an independent risk factor for cardiovascular disease, immune dysfunction, and premature aging (5, 6). Despite the advancement in the understanding of HIV pathomechanism and its therapeutic management, the metabolic and inflammatory consequences remain limited.

Supplementation with Omega-3 polyunsaturated fatty acids, particularly eicosapentaenoic acid and docosahexaenoic acid, has emerged as a widely available, low-cost, and promising agent for metabolic and immune modulation. Recent mechanistic research has shown that omega-3 fatty acids promote the synthesis of specialized pro-resolving lipid mediators, attenuate cytokine production, reduce hepatic triglyceride synthesis, and enhance clearance of triglyceride-rich lipoproteins (7). Clinical investigations in non-HIV populations have also demonstrated consistent reductions in triglycerides and mild anti-inflammatory effects, suggesting therapeutic potential for populations with heightened inflammatory burden (8). Considering documented nutritional inadequacies and the high prevalence of dyslipidemia in patients affected by HIV (9, 10), omega-3 may be a promising agent. However, no comprehensive synthesis of recent randomized trials has systematically evaluated its effects on both metabolic and inflammatory biomarkers in adults living with HIV. This systematic review and meta-analysis was therefore undertaken to address these knowledge gaps by rigorously assessing the impact of omega-3 fatty acid supplementation on lipid profile and inflammatory biomarkers.

## 2 Methods

We conducted this systematic review and meta-analysis accordance with the PRISMA 2020 guidelines (11). A completed PRISMA 2020 checklist is provided in Supplementary File S2. Regarding the eligibility criteria, we considered trials of individuals living with HIV that compared oral omega-3 fatty acid supplementation with placebo, no supplement, or usual care and reported lipid or inflammatory biomarkers of interest. Eligible outcomes were prespecified as apolipoprotein (Apo) A, Apo B, arachidonic acid (AA), total cholesterol, triglycerides, High-Density Lipoprotein HDL-C, LDL-C, Very-Low-Density Lipoprotein (VLDL), CRP, IL-6, and TNF-*α*. We did not prespecify CD4 + T-cell counts or viral load as outcomes, because these viroimmunological measures are predominantly determined by antiretroviral therapy efficacy rather than nutritional supplementation. To ensure exact reproducibility of the supplied analyses, we required investigator-provided numeric triplets for each included outcome (sample size, mean endpoint, or mean change, and SD); trials that did not supply these data were excluded from this study. Randomized intervention trials were eligible when the required numeric data were provided. We placed no language restriction and excluded reviews, case reports, editorials, and articles that did not report the numeric data required for reproduction.

We searched PubMed/MEDLINE, Web of Science, and Scopus databases from 1965 to September 2025. Our search combined controlled vocabulary and keywords for HIV, omega 3 fatty acids, and prespecified biomarkers, using Boolean operators such as (“HIV” OR “HIV infection” OR “human immunodeficiency virus”) AND (“omega-3” OR “EPA” OR “DHA”) AND (“triglycerides” OR “cholesterol” OR “CRP” OR “IL 6” OR “TNF *α*”) AND (“randomized” OR “clinical trial”). Full database-specific search strings are presented in Supplementary Table S1. Reference lists of included studies and relevant reviews were screened for additional reports. We primarily utilized a “leave-one-out” sensitivity analysis, a comprehensive method that recalculates the pooled effect size, excluding a different study each time. This allowed us to identify if any single study, whether high-risk or not, disproportionately skewed the results. Complete, reproducible search strategies for each database are available in the Supplementary Materials.

In the study selection and data extraction stage, search results were imported into reference management software and deduplicated. Two reviewers independently screened titles and abstracts; titles and abstracts flagged by either reviewer were advanced to full-text review. Two reviewers independently assessed full texts for eligibility and resolved disagreements by consensus. For included trials, two reviewers independently extracted study-level data onto a piloted form; extracted items included study identifiers, country, design, sample size, participant characteristics, follow-up duration in months, male participant percentage, and baseline and change-from-baseline means and SDs for each outcome. When a trial reported multiple time points for the same outcome, we used the longest common follow-up across trials for primary analyses; When multiple estimates for a single outcome were reported by a trial, we combined them within-study using published within-study correlation methods to generate a single effect estimate for pooling. All extraction discrepancies were resolved by consensus.

Risk of bias assessment was performed by two reviewers independently using RoB 2, recording domain-level judgments and supporting rationale. The primary pooled analyses included all eligible studies; sensitivity analyses excluded studies judged at high or critical risk of bias. RoB domain counts and overall judgments were considered as potential moderators in subgroup and meta-regression analyses when the number of studies permitted.

For the data synthesis and statistical analysis, continuous outcomes were harmonized to common units (lipids in mmol/L with mg/dL conversions noted; CRP in mg/L; IL-6 and TNF-*α* in pg./mL; apolipoproteins in g/L) and pooled as weighted mean differences using restricted maximum likelihood random-effects models implemented in Stata 17.0. We report pooled weighted mean differences (WMDs) with 95% confidence intervals, *z* tests, and heterogeneity statistics tau^2^, *I*^2^, and *H*^2^. Preplanned subgroup analyses considered follow-up duration categories and percentage male tertiles; meta-regression analyses evaluated percentage male and follow-up duration in months as continuous moderators when study counts allowed. Influence diagnostics included leave-one-out analyses and influence plots. Small-study effects and publication bias were assessed with contour-enhanced funnel plots, Egger’s test, trim- and-fill, and subgroup analyses where feasible.

This systematic review and meta-analysis was not prospectively registered because all objectives, outcomes, and analyses were fully defined after study identification and relied solely on published aggregate data; therefore, prospective registration was not pursued.

## 3 Results

### 3.1 Study selection and study characteristics

**Figure 1:**
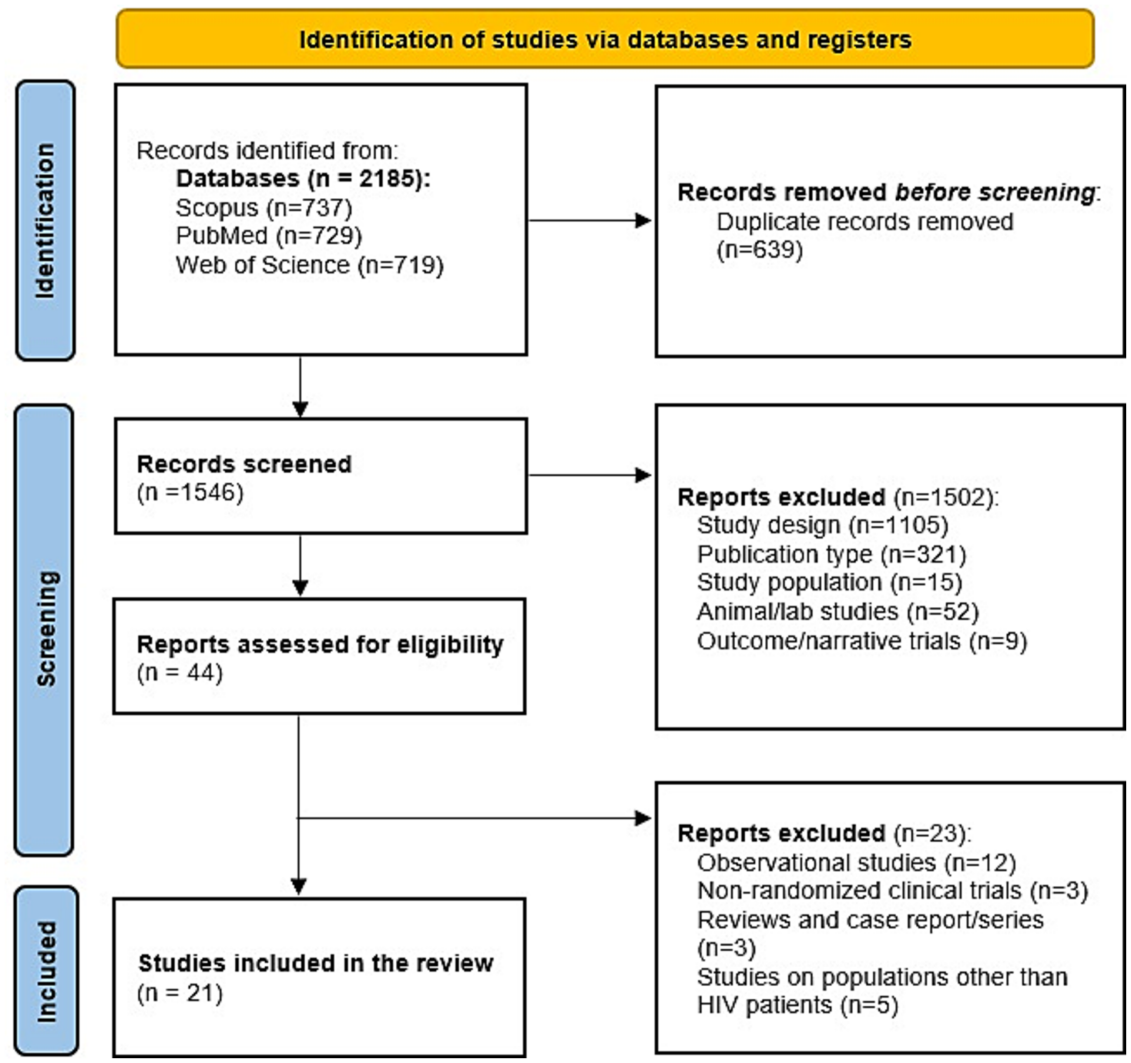
Prisma diagram.

Twenty-one randomized trials representing 1,118 participants met the prespecified inclusion criteria (12–32). The demographic data of each included study are available in Table 1. Pooled outcomes and the number of studies included were as follows: total cholesterol (*n* = 15), HDL-C (*n* = 13), triglycerides (*n* = 13), LDL-C (*n* = 11), CRP (*n* = 8), IL-6 (*n* = 4), Apo A (*n* = 4), and Apo B (*n* = 4). The follow-up period used for primary analyses ranged from 2 to 24 months; the percentage of male participants varied across trials (approximately mid-60s to 100%). All trials compared oral omega-3 supplementation with a placebo or no additional supplement. Detailed study-level characteristics, along with the PRISMA flow diagram, are available in Figure 1. Our leave-one-out analysis confirmed that no single study significantly altered the pooled effect size in all variables, indicating that the results are stable and not driven by outliers. We observed that excluding the subset of high-risk studies did not significantly alter the pooled effect size.

**Table 1:** Characteristics of included studies.

Row	Name	Followup	Criteria	Dosage	Sample size	Male	Age (y)	Years HIV diagnosed	Years of art	CD4 + Count	Dyslipidemia	Inflammatory
1	Amador-Licona et al. (12)	6 months	HIV + on stable HAART (>3 mos.); TG: 2.26 and 5.65 mmol/L; LDL-C: 3.36 and < 4.13 mmol/L; CD4+ > 200 cells.μL	Omega-3 PUFAs: 2.4 g/day (EPA 1200 mg/day+ DHA 600 mg/day)	35	28	39.5 ± 9.5	5.6 ± 2	4.5 ± 1.7	525.7 ± 129.6	TG, VLDL, HDL, and LDL	Lipoperoxides, Glutathione, and Nitric Oxide
	Placebo				30	23	39.9 ± 8	6.8 ± 2.2	5.4 ± 2	663.7 ± 180		
2	Baril et al. (13)	12 months	HIV + adults on stable ART (>6 months); virologically suppressed (VL < 50 copies/mL); fasting TG ≥ 6 mmol/L but <11 mmol/L, or TG > 2 mmol/L and <6 mmol/L with elevated TC/HDL-C on stable lipid-lowering therapy (≥3 months).	Salmon oil omega-3 PUFAs: 3 g/day (EPA 540 mg/day+ DHA 360 mg/day)	26	26	50.9 ± 8.4	9.9 ± 5.2	NA	736 ± 456	TG, TC, HDL-C, LDL-C, and APO B	NA
	Placebo				32	31	47.8 ± 5.5	11.8 ± 5.2	NA	540 ± 307		
3	Capili et al. (14)	8 months	HIV + on PI-ART >6 mo; TG: 1.69–5.65 mmol/L; LDL-C < 3.36 mmol/L; CD4+ > 300 cells/Μl	PUFA 4 g/d (EPA. DHA2400.1600 mg/day)	8	6	46.9 ± 11.5	9.5 ± 6.1	NA	573 ± 284	TG, TC, HDL-C, and LDL-C	NA
	Placebo				10	6	45.6 ± 6.5	12.6 ± 4.9	NA	525 ± 182		
4	de Truchis et al. (15)	8 months	HIV + on stable HAART (>2 mo); TG more than 3.43 mmol/L	Omega-3 PUFAs: 6 g/day (EPA 1080 mg/day+ DHA 720 mg/day)	58	52	45.6 ± 8.6	11 ± 4.5	7.1 ± 2.8	NA	TG, TC, and HDL-C	NA
	Placebo				62	55	47.1 ± 8.4	11.6 ± 4.2	7.7 ± 3.1	NA		
5	Oliveira et al. (16)	24 months	HIV + on stable ART (≥3 mos.); TG > 1.3 mmol/L; LDL-C < 4.14 mmol/L; FPG < 7 mmol/L	Omega-3 PUFAs: 3 g/day (EPA 540 mg/day + DHA 360 mg/day)	63	33	43.1 ± 7.4	10.3 ± 5.7	8.3 ± 4.1	591.8 ± 259.6	TG, LDL, HDL, TC, APO B, APO A1, insulin resistance index, waist circumference, and waist-to-hip ratio (HOMA-IR)	hsCRP
	Placebo				40	31	42.8 ± 6.3	10.9 ± 5	9.2 ± 3.5	616.2 ± 366.9		
6	Paranandi et al. (17)	12 weeks	HIV + on stable HAART (≥3 mos.); TG ≥ 150 mg/dL	Omega-3 PUFAs: 4 g/day (EPA 1860 mg/day+ DHA 1500 mg/day)	41	35	51.7 ± 9.6	16.7 ± 7.2	NA	621.3 ± 277	TG, TC, APO A-1, LDL-C, and HDL-C	NA
	Placebo				41	35	55.0 (44.0–58.0)	16.7 ± 7.2	NA	621.3 ± 277		
7	Peters et al. (18)	12 months	HIV + on stable HAART (>3 mos.); TG: 3.39 & 11.3 mmol/L; on fibrate/niacin therapy	Omega-3 PUFAs: 4 g/day (EPA 1840 mg/day+ DHA 1520 mg/day)	23	23	46.1 ± 2.9	NA	NA	633 ± 217	TG, TC, VLDL, HDL, LDL, APO A1, and APO B100	NA
	Placebo				25	24	43.6 ± 8.9	NA	NA	546 ± 257		
8	Thusgaard et al. (19)	12 months	HIV + on stable ART (≥3 mos.)	Omega-3 PUFAs: 3.6 g/day (EPA 1840 mg/day+ DHA 1520 mg/day)	26	19	43 ± 10	NA	8.1	503 ± 306	TG, TC, HDL-C, LDL-C, APO B, and APO A	LTB4, LTB5, ICAM-1, VCAM-1, and hsCRP
	Placebo				25	21	47 ± 11	NA	8	483 ± 267		
9	Woods et al. (20)	10 months	HIV+; TG > 1.69 mmol/L and.or QUICKI <0.35 or >0.30	Omega-3 PUFAs: 3 g/day (EPA 2000 mg/day+ DHA 1000 mg/day)	28	24	46.2 ± 8.2	NA	NA	527.3 ± 225.2	TG, TC, HDL, LDL, serum phospholipid fatty acids (including EPA, DHA, arachidonic acid, and other fatty acids)	hsCRP
	Placebo				26	19	46.3 ± 5	NA	NA	489.7 ± 228.1		
10	Swanson et al. (21)	3 months	HIV-infected adults aged 40–70 years.hsCRP level > 2.0 mg/L. CD4 + T-cell count ≥ 250 cells.mm/Viral load < 75 copies/mL + . ART for at least 2 months	1.6 g/day (Providing 400 mg EPA + 300 mg DHA per 800 mg gelcap, taken twice daily)	18	9	54.5 ± 5.9	NA	NA	797 ± 602 cells.mm^3^	NA	hsCRP. IL-6. TNF-α. IFN-γ
	Placebo				19	10	53 ± 6.1	NA	NA	672 ± 363 cells.mm^3^		
11	Swanson et al. (22)	12 weeks	HIV-infected adults aged 40–70 years/sCRP level > 3.0 mg/L. CD4 + T-cell count ≥ 250 cells/mm^3^. Viral load < 75 copies/mL. + ARTfor at least 2 months	1 g/day of Krill Oil (Providing 134 mg EPA + 66 mg DHA per day)	5	4	53.4 ± 7.8	NA	NA	626 ± 392 cells.mm^3^	LDL, VLDL, HDL, CHOL, and TG	hsCRP. IL-6. D-dimer
	Placebo				5	3	52.0 ± 4.8	NA	NA	568 ± 77 cells.mm^3^		
12	Hileman et al. (23)	6 months	HIV-1-infected adults. HIV-1 RNA < 400 copies.ml. Stable ART for ≥12 weeks, cumulative ART duration ≥12 months. Framingham 10-year risk score ≥6%. BMI between 19 and 35 kg/m^2^	2 g/day (1 g capsule twice daily of Omega-3-acid ethyl esters. Each 1 g capsule contained ~465 mg EPA and ~365 mg DHA, totaling ~930 mg EPA and ~730 mg DHA per day).	18	18	51	179 ± 71 months	107 ± 50 months	602 ± 277 cells.mm^3^	HDL, LDL, Total CHOL, and TG	hs-CRP. IL-6.sTNFR-I, sTNFR-II. VCAM-1. ICAM-1. D-Dimer. Fibrinogen
	Placebo				17	17	51	140 ± 83 months	12 ± 49 months	604 ± 270 cells.mm^3^		
13	Metkus et al. (24)	2 months	HIV-infected adults. CD4 count > 200 cells.μL. Suppressed viral load (<400 copies.mL). Triglycerides > 200 mg.dL. On stable antiretroviral therapy (ART) for at least 3 months	3.6 g/day of Omega-3-acid ethyl esters (Lovaza). This provided approximately 1.7 g EPA and 1.3 g DHA per day.	24	21	50 (47.0–52.5)	13.3 (10.9–21.0)	9.9 (8.1–11.1)	477 (437–710) cells.μL	TG, TC, HDL, and non-HDL-C	IL-6. TNF-α.hs-CRP.sTNFR-I.sTNFR-II
	Placebo				24	22	48 (41.5–52.5)	13.3 (10.9–21.0)	8.9 (4.5–10.4)	531 (361–661)		
14	Coghil et al. (25)	3 months	HIV and HHV-8 co-infected adults ≥18 years. KS patients had to have early-stage (T0) disease, be on ART for >2 months, and no recent chemotherapy	Fish oil. Daily dose of 3 g	33	18	38 ± 20	NA	NA	Mean 439 (95% CI: 366–513) cells.μl	EHA, DPA, and EPA	IL6 and CRP
	Placebo				34	20	38	NA	NA	Mean 423 (95% CI: 359–487) cells.μL		
15	Domingo et al. (26)	12 months	HIV + patients on stable cART with fasting triglycerides between 2.26–5.65 mmol/L.	Docosahexaenoic acid (DHA) only. Daily dose of 4 g	18	NA	NA	NA	NA	NA	TG, ARA	hsCRP, IL-1, IL-6, IL-8, MCP-1, TNF-α, NGF, and HGF
	Placebo				21	NA	NA	NA	NA	NA		
16	Carter et al. (27)	8 weeks	HIV + males on stable HAART (>6 mos.); Fasting TG: 3.5–10.0 mM; Fasting Chol: ≤6.5 mM	9 g/day (EPA ~ 5,400 mg. DHA ~ 3,600 mg)	5	5	48	NA	NA	393	TG, Cholesterol	NA
	Placebo				6	6	48	NA	NA	393		
17	Wohl et al. (28)	16 weeks	HIV + on ART (≥3 drugs); Fasting TG > 200 mg/dL	~2.9 g/day (EPA. DHA)	26	NA	NA	NA	NA	NA	TG and LDL-C	NA
	Placebo				26	NA	NA	NA	NA	NA		
18	Song et al. (29)	12 weeks	HIV- and HHV8- infected patients in Uganda	Omega-3: 3 g/day (1856 mg EPA + 1,232 mg DHA)	33	NA	NA	NA	NA	NA	Plasma Phospholipid Fatty Acids	NA
	Placebo			High-oleic safflower oil	33	NA	NA	NA	NA	NA		
19	Zhang et al. (30)	12 weeks	HIV+; Age 40–70; hsCRP ≥2.0 mg/L; CD4 + ≥250 cells.mm^3^; VL < 75 copies/mL; stable ART (≥2 months)	Fish Oil: 1.6 g/day (800 mg EPA + 600 mg DHA + 200 mg other omega-3)	18	NA	52	NA	NA	≥250 cells.mm^3^	NA	CD14, LBP, LPS, Zonulin, sCD40L, IP-10, IL-1RA, IFN-α, IL-8, IL-1β, IL-10, GM-CSF, IL-1β, IL-2, IL-2R, IL-4, IL-5, IL-6, IL-7, IL-12, IL-13, IL-15, IL-17, MCP-1, MIG, MIP-1*α*, MIP-1β, RANTES, and TNF-α
	Placebo			Oleic Sunflower Oil: 1.0 g/day	15	NA	52	NA	NA	≥250 cells.mm^3^	NA	
20	Sainz et al. (31)	4 weeks		Symbiotics, omega-3.6, amino acids, 20 g.day	24	9	12 ± 3.9	NA	NA	692 (517–861)	NA	IL-6, IL-7, IP-10, sCD14, IFABP, zonulin, and KT ratio
	Placebo							NA	NA	692 (517–861)	NA	
21	Dong et al. (32)	6 months	HIV-1 infected, stable cART (>6 months), diagnosed with Neurocognitive Impairment (NCI), BMI ≤ 30 kg/m^2^, no major comorbidities	DHA algae oil: 3.15 g/day	35	30	54.6 ± 9.7	NA	NA	381.0 (226.0, 518.0)	TG, TC, HDL, and LDL	TNF-α, IL-6, sCD14, and hs-CRP
	Placebo			Placebo (soy oil)	35	26	55.3 ± 9.3	NA	NA	387.0 (293.0, 593.0)		

### 3.2 Inflammatory biomarkers

**Figure 2:**
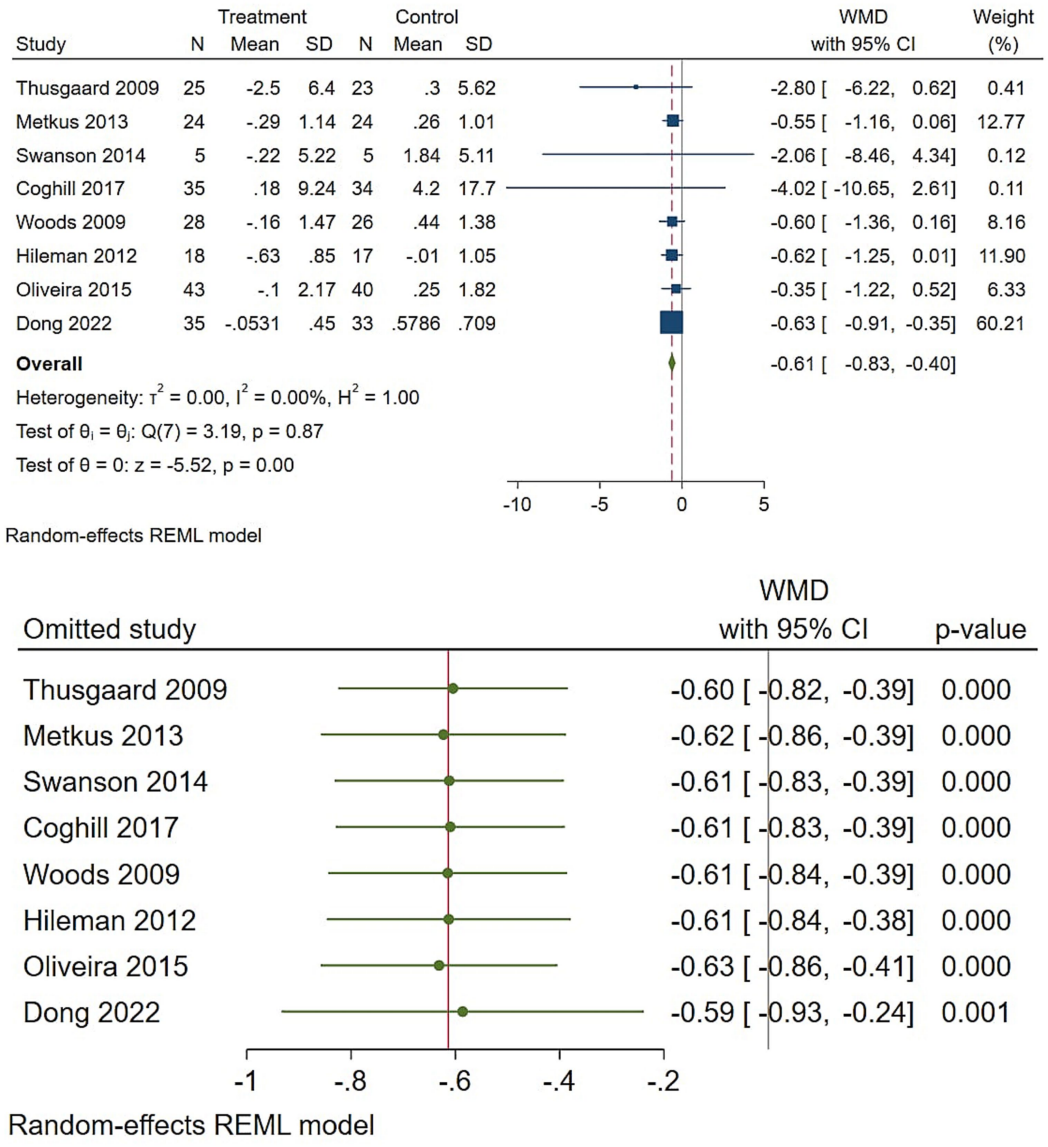
Effect of omega-3 supplementation on the serum level of CRP in HIV-positive patients. Up: Forest plot; Down: Sensitivity analysis.

Regarding the CRP, across eight trials, omega-3 supplementation produced a small but consistent reduction in CRP (Figure 2). The reproduced random-effects (REML) pooled WMD was −0.61 mg/L (95% CI −0.83 to −0.40; z *p* < 0.001), with essentially no between-study heterogeneity (tau2 = 0.000; I2 = 0%). Results were robust when leave-one-out sensitivity checks were performed (Figure 2) and a trim-and-fill adjustment produced a similar result (observed −0.61; observed + imputed −0.60 (Supplementary file). In plain terms, omega-3 s reliably lowered CRP by approximately 0.6 mg/L in these trials; although the changes were modest, the consistency of this effect across studies further indicated that it reflects a real anti-inflammatory signal.

**Figure 3:**
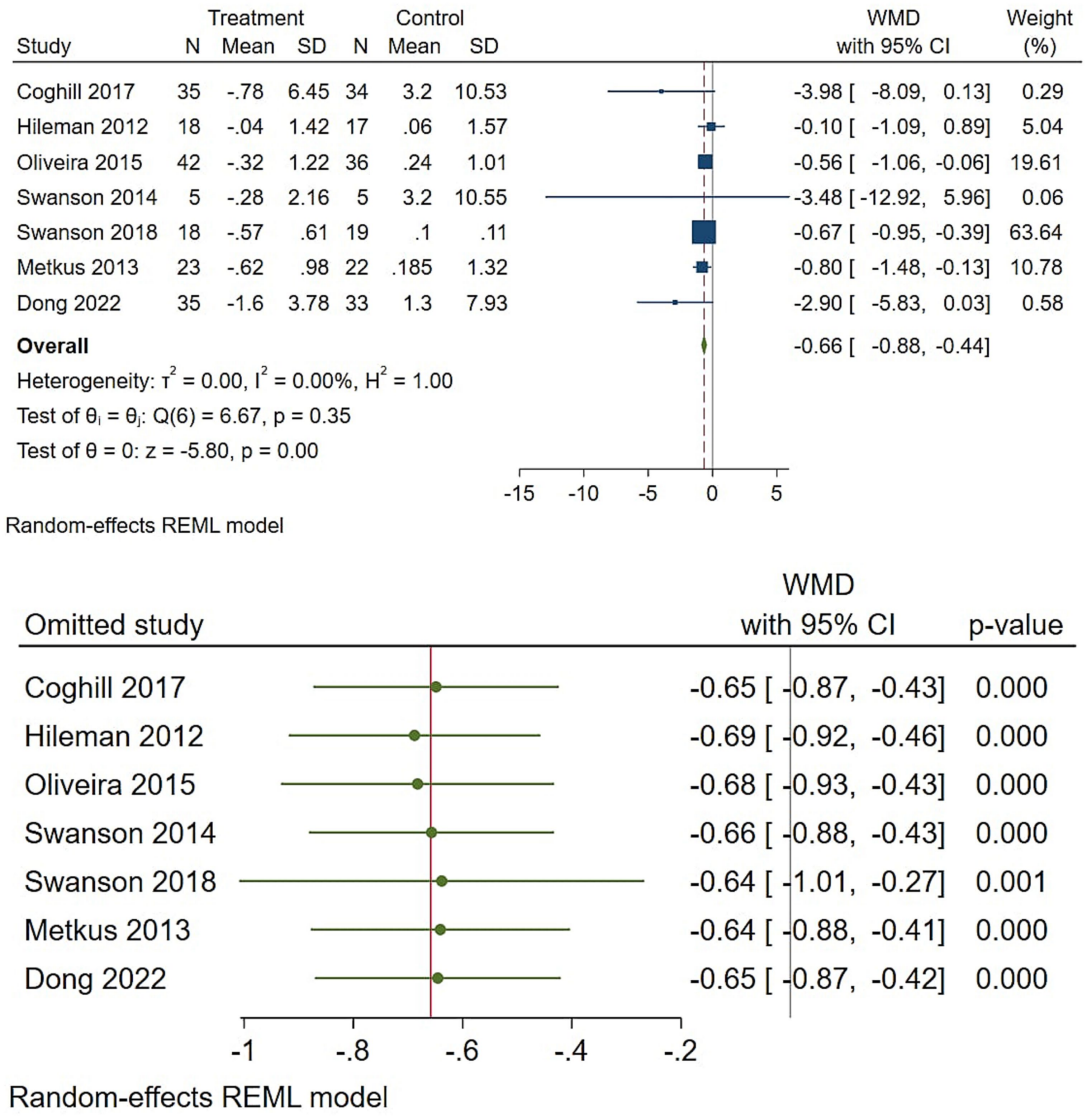
Effect of omega-3 supplementation on the serum level of IL-6 in HIV-positive patients. Up: Forest plot; Down: Sensitivity analysis.

Regarding IL-6, four trials contributed to the IL-6 pooled estimate, which favored omega-3 but with less precision than CRP (Figure 3). The reproduced WMD was −0.66 pg./mL (95% CI −0.88 to −0.44; *p* = 0.048), and the heterogeneity was low (tau2 ≈ 0.05; I2 ≈ 0%). Sensitivity checks showed that the negative direction persisted when individual studies were omitted, and a trim-and-fill adjustment produced a similar result (Figure 3). Because the confidence interval only narrowly excludes no effect and fewer studies contributed data, this finding is suggestive of a significant IL-6 reduction but should be interpreted cautiously.

For other inflammatory biomarkers, such as TNF-alpha, the pooled estimate (Hedges’s g −2.06, 95% CI −4.35 to 0.24; *n* = 3) suggests a large effect; however, this analysis was based on only three small and highly heterogeneous trials (I2 ≈ 97%) using differing assays and units. Therefore, TNF-*α* findings are presented as exploratory and no firm conclusions could be drawn. The three trials are small and yield markedly different answers (I2 ≈ 97%), such that a single study can alter the pooled conclusion, and small-study effects are likely. In addition, variations in measurement and reporting methods across trials may indicate that the standardized result may likely combine incompatible data. Taken together, the inflammatory results point to a modest anti-inflammatory effect of omega-3 supplementation in adults with HIV. The evidence is clearest for CRP (precise and consistent); IL-6 shows a similar but smaller and less certain signal.

### 3.3 Lipid profiles

**Figure 4:**
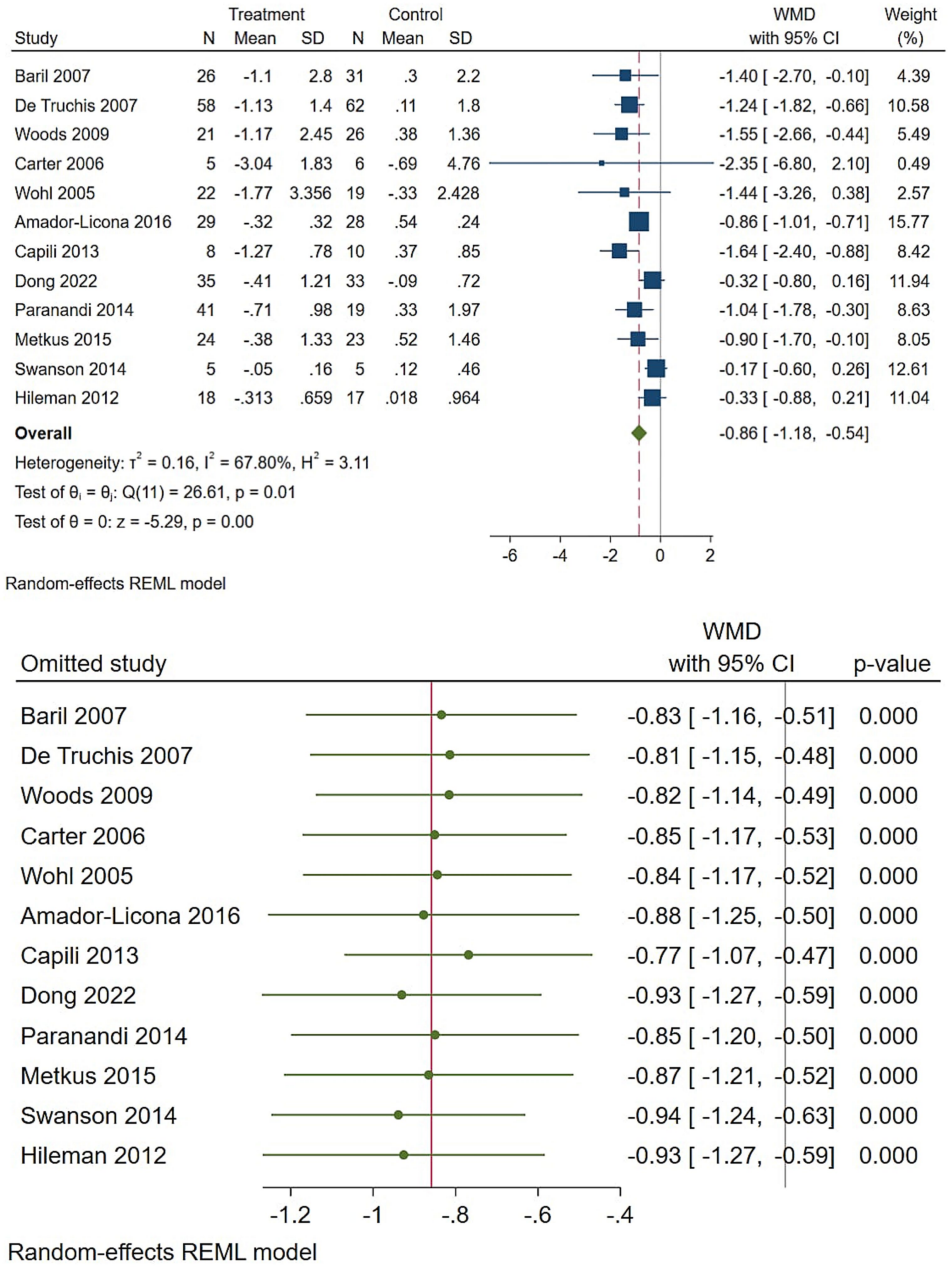
Effect of omega-3 supplementation on the serum level of triglycerides in HIV-positive patients. Up: Forest plot; Down: Sensitivity analysis.

Regarding the triglycerides, omega-3 supplementation produced a clear and clinically meaningful reduction in triglycerides (Figure 4). The pooled random-effects WMD was −0.86 (95% CI −1.18 to −0.54), indicating a statistically significant effect that remained robust in leave-one-out and influence checks, despite substantial heterogeneity (I2 ≈ 67%). The directions and magnitudes of these effects are clinically plausible and likely most relevant for patients with elevated baseline triglyceride levels.

**Figure 5:**
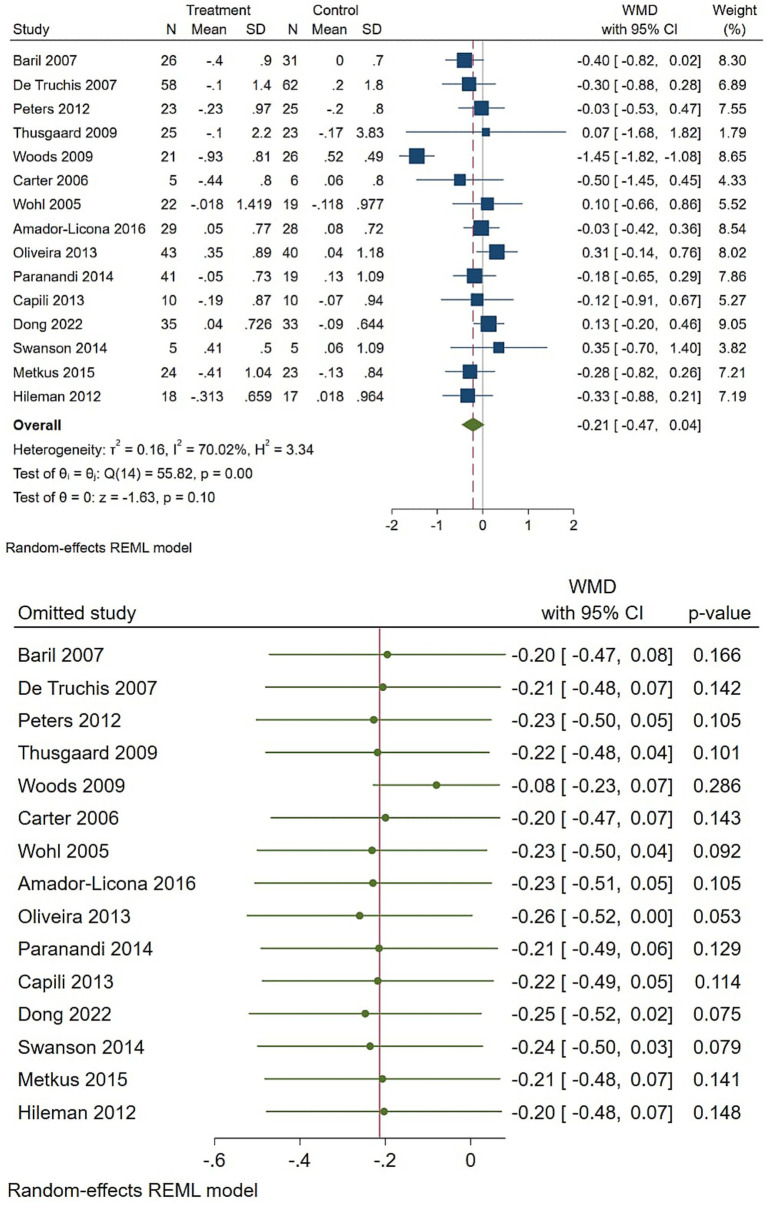
Effect of omega-3 supplementation on the serum level of total cholesterol in HIV-positive patients. Up: Forest plot; Down: Sensitivity analysis.

Total cholesterol showed a small, non-significant reduction, with a pooled WMD of −0.21 (95% CI −0.47 to 0.04) (Figure 5). Heterogeneity was substantial (I2 ≈ 70%), which reduces confidence in a true effect and suggests that there is a potential for between-study differences in population, dose, or follow-up to explain some variation. The estimate is imprecise and should be interpreted cautiously.

**Figure 6:**
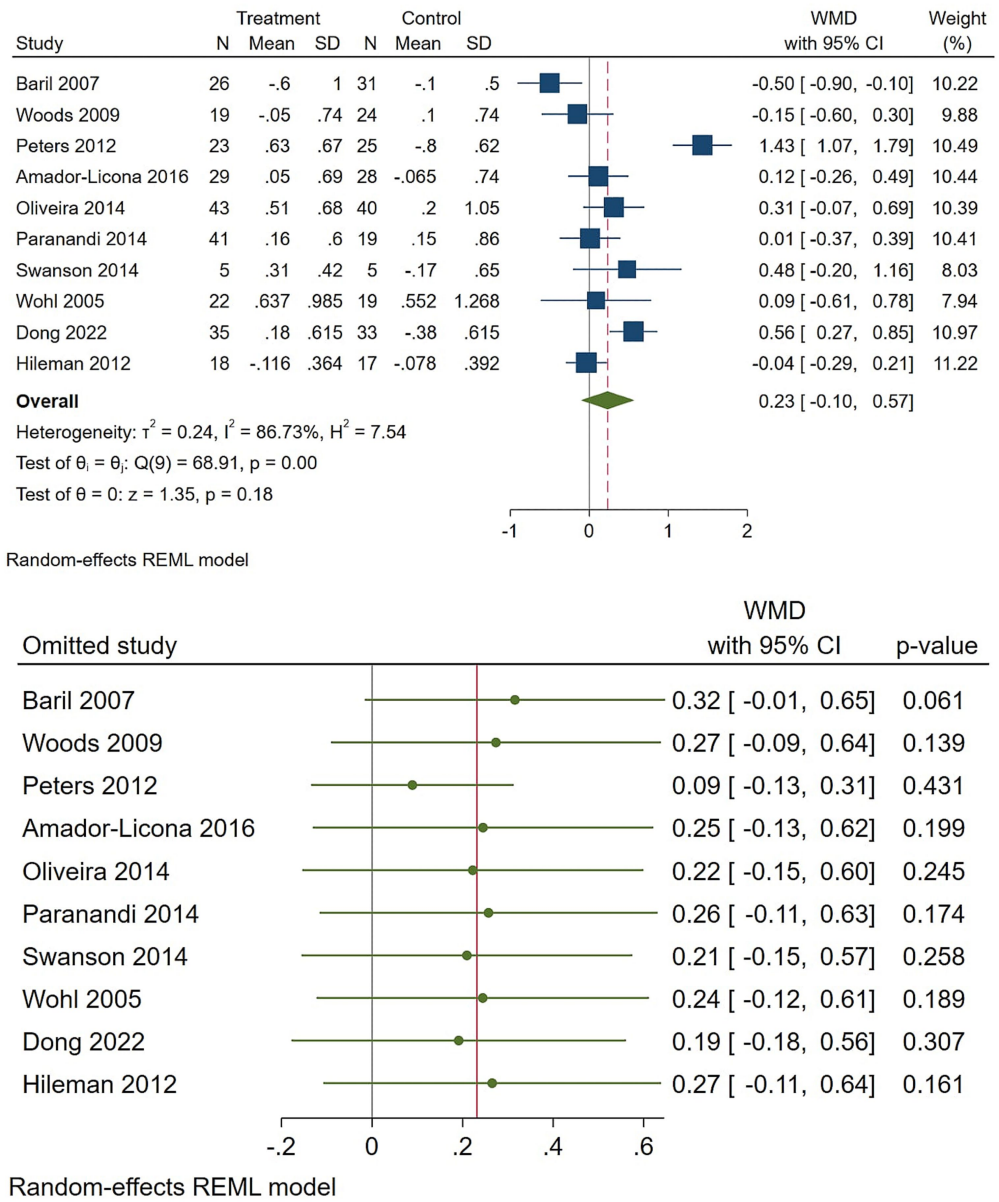
Effect of omega-3 supplementation on the serum level of LDL cholesterol in HIV-positive patients. Up: Forest plot; Down: Sensitivity analysis.

**Figure 7:**
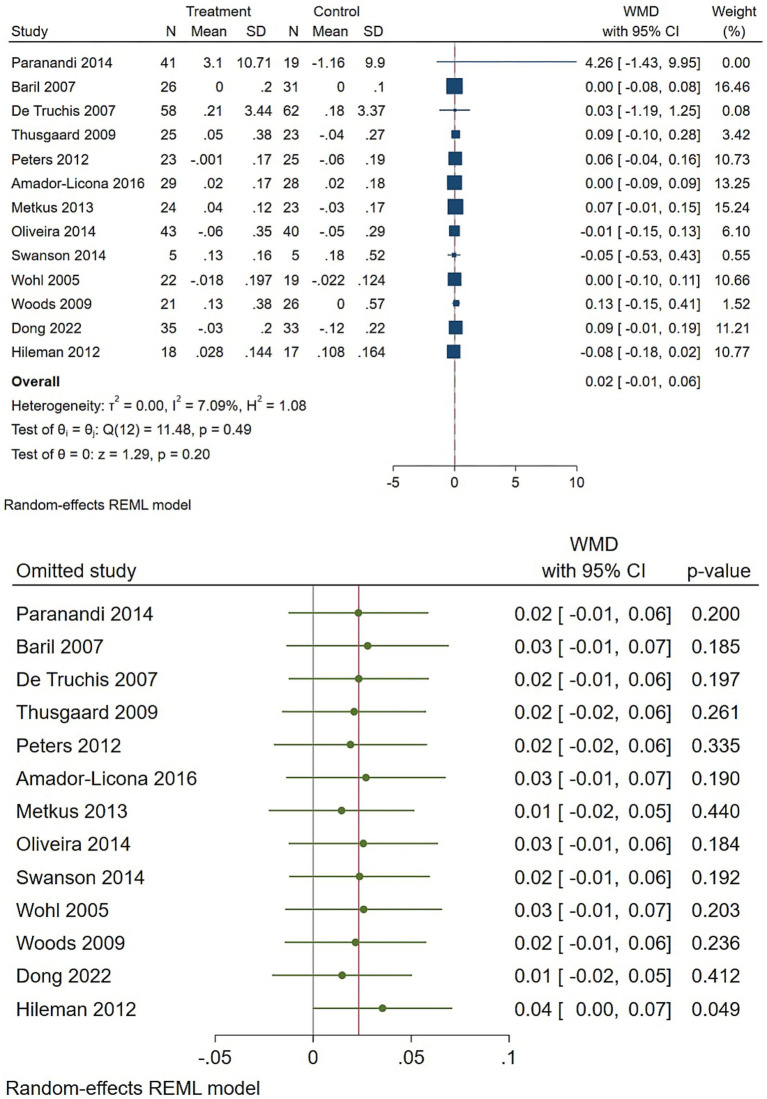
Effect of omega-3 supplementation on the serum level of HDL cholesterol in HIV-positive patients. Up: Forest plot; Down: Sensitivity analysis.

For LDL-C, results were inconsistent and imprecise. The pooled WMD was 0.23 (95% CI −0.10 to 0.57) with very high heterogeneity (I2 ≈ 87%), indicating marked variation across trials and no clear directional effect (Figure 6). Sensitivity and subgroup checks indicated that study characteristics influenced results; the overall LDL-C finding is therefore inconclusive. In addition, HDL-C showed a trivial, non-significant pooled increase (WMD 0.02; 95% CI −0.01 to 0.06) with negligible heterogeneity (I2 = 7%), indicating consistently minimal effect across studies. Sensitivity analyses did not modify this conclusion (Figure 7).

**Figure 8:**
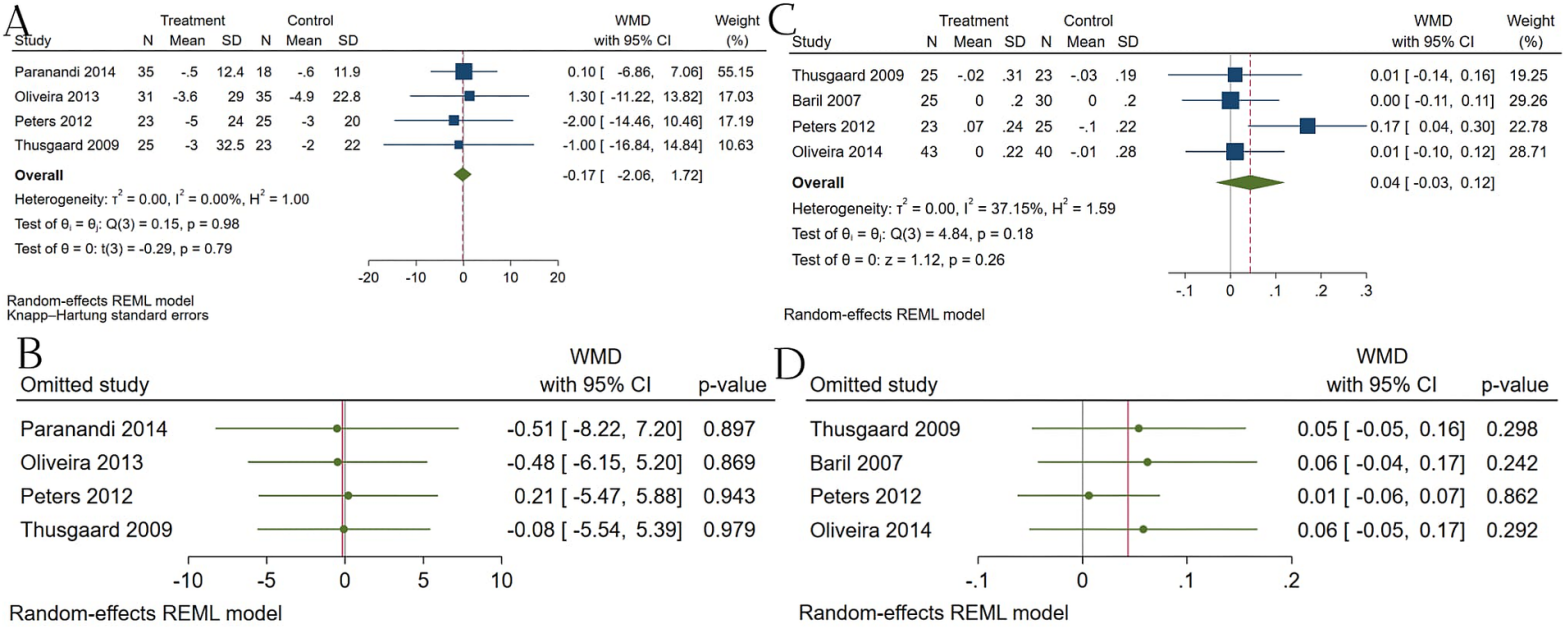
Effect of omega-3 supplementation on the serum level of apolipoprotein in HIV-positive patients. **(A)** Forest plot of the effect of omega-3 on apolipoprotein A; **(B) s**ensitivity analysis of the effect of omega-3 on apolipoprotein A; **(C)** forest plot of the effect of omega-3 on apolipoprotein B; **(D)** sensitivity analysis of the effect of omega-3 on apolipoprotein B.

Omega-3 showed no significant effect on apolipoproteins. Among apolipoproteins, Apo B pooled to 0.04 (95% CI −0.03 to 0.12) with low–moderate heterogeneity (I2 ≈ 37%), showing no clear effect. Apo A pooled to −0.17 (95% CI −5.34 to 4.99) with no heterogeneity but very wide confidence intervals due to sparse data, rendering the estimate uninformative. Sensitivity checks did not reveal hidden large effects for Apo B; Apo A remains underpowered (Figure 8).

Among lipid factors, the arachidonic acid and VLDL produced large standardized reductions in pooled analyses, but each estimate rests on only two small, methodologically heterogeneous trials. AA measurements were gathered from different specimen types and were reported as percentages or absolute concentrations; some VLDL values were indirectly derived from triglycerides or provided in incompatible units. Because single studies drive the results and biological interpretation is obscured by metric differences, these findings should be presented as hypothesis-generating, concise study-level details (matrix, unit, endpoint/change, dose, N, direction) should be reported, and larger, standardized trials before drawing clinical conclusions should be recommend.

Overall, the lipid data point to a robust and clinically relevant triglyceride reduction with omega-3 supplementation; total cholesterol and LDL-C findings are inconsistent and imprecise, HDL-C changes are negligible, and apolipoprotein results are null or underpowered. Triglycerides represent the clearest lipid benefit, while other lipid outcomes require cautious interpretation given heterogeneity and limited precision.

### 3.4 Subgroup analysis

Subgroup results are shown in Table 2. Total cholesterol exhibited significant subgroup differences by both follow-up duration and sex composition (*p* for subgroup = 0.00 for both). Notably, trials with 7–12 months follow-up showed a larger (more negative) WMD (−0.23 mmol/L) and high within-subgroup heterogeneity (I2 = 72%), whereas trials with >12 months had a positive WMD (0.31 mmol/L). Based on sex composition, total cholesterol estimates became more negative as the percent male increased (WMDs: <75% = −0.09; 75–90% = −0.30; >90% = −0.37 mmol/L). Triglycerides showed a significant difference across percentage-male strata (*p* = 0.004) but not by follow-up duration (*p* = 0.163). The largest reductions were observed in studies with 75–90% male (WMD −1.04 mmol/L; I2 = 60%) and in those with <75% male (WMD −0.86 mmol/L; I2 = 0%), whereas trials with >90% male had smaller, non-significant reductions (WMD −0.33 mmol/L). LDL-C subgroup testing indicated a significant difference by male participant percentage (*p* = 0.00) but not by follow-up duration (*p* = 0.639). The >90% male subgroup contained a single study with a large positive WMD (1.43 mmol/L), while the 75–90% male subgroup showed an imprecise positive estimate (WMD 0.24; I2 = 88.8%) and the <75% male subgroup showed a null effect (WMD 0.12; I2 = 0%).

**Table 2:** Results of subgroup analysis of included studies.

Outcome	Subgroup_variable	Subgroup_level	N	N_participant_total	Pooled_metric	Pooled_estimate	CI_lower	CI_upper	I2	tau2	Model	p_for_subgroup
Total cholesterol	FollowUp_months	0–3 months	4	151	WMD (mmol/L)	−0.13	−0.33	0.07	0	0	REML	0
Total cholesterol	FollowUp_months	4–6 months	3	171	WMD (mmol/L)	−0.13	−0.37	0.11	7.6	0.01	REML	0
Total cholesterol	FollowUp_months	7–12 months	3	206	WMD (mmol/L)	−0.23	−0.57	0.11	72.4	0.19	REML	0
Total cholesterol	FollowUp_months	>12 months	2	95	WMD (mmol/L)	0.31	−0.14	0.76	0	0	REML	0
Total cholesterol	PercentMale	<75% male	5	223	WMD (mmol/L)	−0.09	−0.4	0.22	45	0.09	REML	0
Total cholesterol	PercentMale	75–90% male	4	240	WMD (mmol/L)	−0.3	−0.7	0.1	68	0.15	REML	0
Total cholesterol	PercentMale	>90% male	3	160	WMD (mmol/L)	−0.37	−0.85	0.11	55	0.12	REML	0
Triglycerides	FollowUp_months	0–3 months	3	110	WMD (mmol/L)	−0.55	−1.4	0.3	74.7	0.28	REML	0.163
Triglycerides	FollowUp_months	4–6 months	3	160	WMD (mmol/L)	−0.66	−1.16	−0.16	70.4	0.1	REML	0.163
Triglycerides	FollowUp_months	7–12 months	4	210	WMD (mmol/L)	−1.39	−1.85	−0.93	0	0	REML	0.163
Triglycerides	PercentMale	<75% male	3	155	WMD (mmol/L)	−0.86	−1.01	−0.71	0	0	REML	0.004
Triglycerides	PercentMale	75–90% male	5	260	WMD (mmol/L)	−1.04	−1.78	−0.3	60	0.12	REML	0.004
Triglycerides	PercentMale	>90% male	3	141	WMD (mmol/L)	−0.33	−0.88	0.21	0	0	REML	0.004
LDL-C	FollowUp_months	0–3 months	2	120	WMD (mmol/L)	0.16	−0.27	0.58	29.1	0.03	REML	0.639
LDL-C	FollowUp_months	4–6 months	1	57	WMD (mmol/L)	0.12	−0.26	0.49	0	0	REML	0.639
LDL-C	FollowUp_months	7–12 months	3	150	WMD (mmol/L)	0.47	−1.43	2.36	97.9	1.82	REML	0.639
LDL-C	FollowUp_months	>12 months	1	43	WMD (mmol/L)	0.31	−0.07	0.69	0	0	REML	0.639
LDL-C	PercentMale	<75% male	3	210	WMD (mmol/L)	0.12	−0.26	0.49	0	0	REML	0
LDL-C	PercentMale	75–90% male	3	120	WMD (mmol/L)	0.24	−0.23	0.71	88.8	0.35	REML	0
LDL-C	PercentMale	>90% male	1	76	WMD (mmol/L)	1.43	1.07	1.79	NA	NA	REML	0
HDL-C	FollowUp_months	0–3 months	3	90	WMD (mg/dL)	0.07	−0.01	0.15	54.3	0.05	REML	0.845
HDL-C	FollowUp_months	4–6 months	1	57	WMD (mg/dL)	0	−0.09	0.09	0	0	REML	0.845
HDL-C	FollowUp_months	7–12 months	3	210	WMD (mg/dL)	0.03	−0.03	0.09	0	0	REML	0.845
HDL-C	PercentMale	<75% male	4	180	WMD (mg/dL)	0	−0.05	0.05	0	0	REML	0.738
HDL-C	PercentMale	75–90% male	3	190	WMD (mg/dL)	0.06	−0.04	0.16	0	0	REML	0.738
HDL-C	PercentMale	>90% male	2	124	WMD (mg/dL)	0.09	−0.1	0.28	0	0	REML	0.738
IL-6	FollowUp_months	0–3 months	2	116	WMD (pg/mL)	−3.9	−7.67	−0.13	0	0	REML	0.12
IL-6	FollowUp_months	4–6 months	1	18	WMD (pg/mL)	−0.1	−1.09	0.89	0	0	REML	0.12
IL-6	PercentMale	<75% male	1	35	WMD (pg/mL)	−3.98	−8.09	0.13	NA	NA	REML	0.235
IL-6	PercentMale	75–90% male	1	23	WMD (pg/mL)	−0.8	−1.48	−0.13	NA	NA	REML	0.235
IL-6	PercentMale	>90% male	2	101	WMD (pg/mL)	−0.1	−3.9	3.7	9.2	0.05	REML	0.235
CRP	FollowUp_months	0–3 months	3	88	WMD (mg/L)	−3.01	−7.61	1.6	0	0	REML	0.452
CRP	FollowUp_months	4–6 months	2	42	WMD (mg/L)	−0.59	−1.18	0	0	0	REML	0.452
CRP	FollowUp_months	7–12 months	2	80	WMD (mg/L)	−2.8	−6.22	0.62	0	0	REML	0.452
CRP	PercentMale	<75% male	2	70	WMD (mg/L)	−2.06	−8.46	4.34	NA	NA	REML	0.591
CRP	PercentMale	75–90% male	2	96	WMD (mg/L)	−0.58	−1.16	−0.01	0	0	REML	0.591
CRP	PercentMale	>90% male	1	44	WMD (mg/L)	−0.62	−1.25	0.01	NA	NA	REML	0.591
ApoA	FollowUp_months	0–3 months	1	35	WMD (mg/dL)	0.1	−6.86	7.06	NA	NA	REML	0.537
ApoA	FollowUp_months	7–12 months	2	48	WMD (mg/dL)	−1.5	−11.41	8.18	0	0	REML	0.537
ApoA	PercentMale	<75% male	2	66	WMD (mg/dL)	0.6	−11.22	12.42	NA	NA	REML	0.184
ApoA	PercentMale	75–90% male	1	23	WMD (mg/dL)	−2	−14.46	10.46	NA	NA	REML	0.184
ApoB	FollowUp_months	12 months	3	73	WMD (g/L)	0.06	−0.05	0.17	54.4	0	REML	0.537
ApoB	FollowUp_months	24 months	1	83	WMD (g/L)	0.01	−0.1	0.12	NA	NA	REML	0.537
ApoB	PercentMale	<75% male	2	86	WMD (g/L)	0.01	−0.1	0.12	NA	NA	REML	0.184
ApoB	PercentMale	>90% male	2	148	WMD (g/L)	0.07	−0.03	0.17	37.2	0	REML	0.184

For HDL-C, CRP, IL-6, Apo A, and Apo B, there were no statistically significant subgroup differences by follow-up or percentage male; estimates for these outcomes were generally consistent across strata (for example, HDL-C WMDs clustered around 0.00–0.09 mg/dL with I2 ≈ 0%, and CRP WMDs were negative but did not differ significantly between subgroups).

In sum, the table shows that sex composition (percentage male)—and to a lesser extent follow-up duration—helps explain heterogeneity for some lipid outcomes (total cholesterol, LDL-C, and triglycerides). Inflammatory markers and HDL-C were broadly consistent across the examined subgroups. The exact estimates, confidence intervals, heterogeneity statistics, and *p*-values are presented in the submitted subgroup table.

### 3.5 Publication bias and small-study effects

We assessed small-study effects using Egger’s regression for outcomes with available test outputs and complemented these with trim-and-fill sensitivity analyses reported in the analysis logs, Supplementary Figures S1–S7. Egger’s tests were not significant for Apo B (beta1 = 2.94; SE = 5.20; z = 0.57; *p* = 0.572), Apo A (beta1 = −0.20; SE = 1.57; z = −0.13; *p* = 0.898), HDL-C (beta1 = 0.57; SE = 0.56; z = 1.02; *p* = 0.309), LDL-C (beta1 = 0.13; SE = 5.10; z = 0.02; *p* = 0.980), and total cholesterol (beta1 = 0.74; SE = 1.05; z = 0.70; *p* = 0.482). Triglycerides showed a borderline but not significant Egger’s test result (beta1 = −1.25; SE = 0.70; z = −1.77; *p* = 0.076), which may reflect a possible small-study effect and warrants careful consideration. For inflammatory markers, Egger’s test was non-significant for CRP (beta1 = −0.59; SE = 0.543; z = −1.09; *p* = 0.275) and for IL-6 (beta1 = −0.84; SE = 0.558; z = −1.50; *p* = 0.133). Trim-and-fill produced minimal adjustments for most outcomes (Apo B: 0 studies imputed; CRP and IL-6: 2 imputed with negligible change to pooled WMDs; HDL-C, LDL-C, and TG: 0–2 imputed with trivial effect on estimates) but imputed five studies for total cholesterol and shifted the pooled WMD from −0.21 (95% CI −0.47 to 0.04) to −0.38 (95% CI −0.62 to −0.13). Because several outcomes include relatively few trials and some analyses show substantial heterogeneity, Egger’s test is underpowered in those settings; overall, there is limited consistent evidence of publication bias, though the borderline Egger’s test result for triglycerides and the trim-and-fill shift for total cholesterol should be noted in the discussion. The detailed results for subgroup analysis are shown in Table 2.

### 3.6 Quality control

The quality control was checked using Version 2 of the Cochrane risk-of-bias tool for randomized trials (RoB 2), which consists of five domains, designed for quality assessment in randomized clinical trials. The results for quality assessment of the included studies are presented in Table 3.

**Table 3:** Results of quality control of included studies.

Number	Author, Year	Sequence generation	Allocation concealment	Blinding of participants, personnel, and outcome assessment	Incomplete outcome data	Selective outcome reporting	Other potential threats to validity
1	Amador-Licona et al. (12), 2016	L	L	L	L	L	L
2	Baril et al. (13), 2007	L	L	L	H	L	H
3	Capili et al. (14), 2013	L	L	L	H	H	U
4	de Truchis et al. (15), 2006	L	L	L	H	H	U
5	Oliveira et al. (16), 2013	L	L	U	L	L	L
6	Paranandi et al. (17), 2014	U	U	L	L	L	L
7	Peters et al. (18), 2012	L	L	L	L	L	H
8	Thusgaard et al. (19), 2009	L	L	L	L	L	L
9	Woods et al. (20), 2009	U	U	H	L	U	U
10	Swanson et al. (21), 2018	L	L	L	L	L	L
11	Swanson et al. (22), 2014	L	L	L	L	L	L
12	Hileman et al. (23), 2012	L	L	L	L	L	L
13	Metkus et al. (24), 2013	L	L	L	L	L	L
14	Coghil et al. (25), 2017	L	L	L	L	L	L
15	Domingo et al. (26), 2018	L	L	L	L	L	L
16	Carter et al. (27), 2006	L	L	L	L	L	L
17	Wohl et al. (28), 2005	U	U	H	L	L	U
18	Song et al. (29), 2016	L	L	L	L	L	L
19	Zhang et al.(30), 2018	L	L	L	L	L	L
20	Sainz et al. (31), 2022	L	U	L	L	U	L
21	Dong et al. (32), 2022	L	L	L	L	L	L

L, low risk of bias; H, high risk of bias; U, unclear risk of bias.

## 4 Discussion

The remarkable increase in longevity among HIV patients has shifted clinical focus toward the long-term management of iatrogenic metabolic disturbances, particularly those arising from antiretroviral therapy (ART). Against this backdrop, and informed by extensive evidence on the lipid-modifying and anti-inflammatory properties of omega-3, this systematic review and meta-analysis was conducted to elucidate the efficacy of omega-3 supplementation in modulating metabolic parameters and inflammatory biomarkers in individuals affected by HIV.

Based on the results of the current review, treatment with omega-3 seems to exert a favorable effect on CRP and with less significance on the pro-inflammatory response by IL-6. This anti-inflammatory effect of omega-3 was previously suggested in other chronic conditions, such as coronary disease, rheumatoid arthritis, inflammatory bowel disease, asthma, and chronic kidney disease. (33–37); however, for HIV patients, the number of evidence-based studies is limited. The topic is of clinical significance, since suboptimal intake of nutrients, including omega-3, is quite common among patients with HIV on ART (9). Omega-3 fatty acids are converted to anti-inflammatory eicosanoids, which decrease the expression of adhesion molecules within the endothelium, disturbing the chemotactic activity of cytokine-producing leukocytes (38). Although omega-3 s propose an optimal, widely available anti-inflammatory treatment, further clinical trials are expected to investigate their long-term safety and efficacy.

In this study, alterations in the lipid data point to a robust effect of omega-3 supplementation on triglyceride reduction, while for the total cholesterol and LDL-C, the findings were inconsistent. Along with the degenerative changes associated with aging in HIV patients, several studies have recommened that ART may contribute to altered cardiovascular biomarkers, such as lipid profiles [reviewed in (39)], with recommendations provided for switching to an NNRTI (Non-Nucleoside Reverse Transcriptase Inhibitor)-containing antiretroviral regimen. In parallel with our study, omega-3 supplementation, as fish oil or part of the Mediterranean regimen, has been introduced as an adjuvant treatment for TG-lowering and cardioprotection purposes (40). Omega-3 appears to influence the level of TG through several mechanisms, including reduction of plasma FA, directing the metabolism toward phospholipid synthesis, decreasing the TG-producing enzymes, and accelerating the blood clearance of TG-rich particles (41).

Using a GRADE-informed approach, the certainty of evidence for triglyceride reduction was judged as moderate (multiple RCTs, consistent direction, and some heterogeneity), for CRP as moderate (low heterogeneity and modest effect size), and for IL-6 as low (few trials, borderline confidence intervals). Evidence for total cholesterol, LDL-C, HDL-C, and TNF-*α* was graded as low or very low because of substantial heterogeneity, imprecision, small numbers of trials, or exploratory analyses.

Our subgroup analysis revealed a male preponderance in omega-3-related reduction of cholesterol, triglyceride, and LDL. This difference may be attributed to the interplay between the estrogen hormone and omega-3 FAs in terms of hepatic lipid metabolism. A growing body of evidence suggests that the potent hypolipidemic effects of estrogen, particularly its upregulation of hepatic LDL receptor expression and activity, can modulate the lipid-modifying outcomes of omega-3 supplementation (42, 43). This interaction, therefore, may cause the dominant signaling pathways activated by estrogen to mitigate or obscure the observable effects of omega-3 FAs on specific lipid parameters, most notably LDL (44). Consequently, the efficacy of omega-3 supplementation cannot be viewed in isolation but must be considered within the context of the individual’s endocrine milieu and baseline lipid profile, which might influence the response to nutritional interventions. In addition, because these analyses are ecological and the differences are modest, they should be interpreted with caution. The majority of included trials enrolled predominantly male participants, consistent with historical patterns in HIV research. The percentage of male participants (percentage male) ranged from roughly two-thirds to almost exclusively male across studies. Therefore, the apparent male preponderance likely reflects a combination of sex-hormone-dependent lipid metabolism and sampling patterns, and it underlines the need for future trials to report sex-stratified results to clarify true sex differences.

Besides ART, ongoing immune activation and direct impacts of the virus on immune manipulation in people living with HIV causes a persistent inflammation in these patients (45, 46). It is shown that retroviruses exert their inflammatory effect via hijacking immune cells and skewing immune balance (47, 48). This constant immune activation contributes to a wide range of complications across multiple organs in livers, bone, renal, and cardiovascular systems (49). Interestingly, this constant inflammation promotes further metabolic consequences by insulin resistance and disturbed lipid metabolism (50). Our results revealed that omega-3 could reduce inflammatory biomarkers. Therefore, clinicians should consider prescribing omega-3 in people living with HIV to reduce inflammation and its further consequences. Current HIV treatment guidelines (e.g., DHHS and NIH/CDC) emphasize comprehensive cardiovascular risk management, including dyslipidemia control and reduction of chronic inflammation in people living with HIV (51). Our findings, particularly the consistent triglyceride-lowering effect, support considering omega-3 supplementation as a low-cost adjunctive strategy alongside statins, lifestyle modification, and ART optimization in patients with hypertriglyceridemia.

## 5 Conclusion

In adults with HIV, omega-3 supplementation was associated with small, insignificant increases in HDL-C and meaningful reductions in triglycerides, whereas effects on other lipid fractions were inconsistent. Omega-3 supplementation was also associated with a consistent reduction in CRP and modest improvements in other inflammatory biomarkers such as IL-6, whereas evidence for TNF-*α* remains inconclusive. These findings support the beneficial effect of omega-3 supplementation along with standard therapy in patients with HIV infection, for maintaining the lipid and inflammatory profile in normal status. However, guideline-level recommendations will require additional large, long-term trials specifically designed for HIV populations.

## 6 Limitations

This study fills a critical knowledge gap in elucidating the association between omega-3 administration and HIV inflammatory and metabolic factors; however, there were several limitations that need to be considered. First, some variations existed across studies despite our efforts to identify the sources of heterogeneity. Second, the amount of evidence was limited for some variables, hindering us from drawing any conclusion on the causality. Our subgroup analyses were limited to follow-up duration in months and male participant percentage because these variables were consistently reported across trials. Clinically important factors such as ART regimen, HIV stage, baseline dyslipidemia, and omega-3 dosage or formulation were reported too inconsistently to allow credible subgroup meta-analyses. We therefore view the current subgroup findings as exploratory. Future randomized trials should report and predefine these variables to permit more clinically relevant stratified analyses. Therefore, for an optimal metabolic and inflammatory assessment of omega-3, further efforts on prospective long-term studies are warranted in the future.

## Data Availability

The original contributions presented in the study are included in the article/Supplementary material, further inquiries can be directed to the corresponding author.
