# Supplementary material for: Effect of omega-3 supplementation on metabolic and inflammatory markers in adults with HIV infection: a systematic review and meta-analysis
Source: Front Nutr. 2026 Mar 10;13:1746723. doi: 10.3389/fnut.2026.1746723 (PMC13008728; doi:10.3389/fnut.2026.1746723)
Supplement: Supplementary file 1 [file Table_1.docx]

**Supplementary file**

1. Search strategy

**Pubmed: 356 results**

**Pubmed search :**

("C-reactive protein"[All Fields] OR "CRP"[All Fields] OR "ESR"[All Fields] OR "Erythrocyte sedimentation rate"[All Fields] OR "tumor necrosis factor"[All Fields] OR "TNF"[All Fields] OR "tumour necrosis factor alpha"[All Fields] OR "TNF-alpha"[All Fields] OR "interleukin"[All Fields] OR "Inflammation"[All Fields] OR "inflammatory markers"[All Fields] OR "inflammatory biomarkers"[All Fields] OR "IL1"[All Fields] OR "IL-1"[All Fields] OR "interleukin-1"[All Fields] OR "IL2"[All Fields] OR "IL-2"[All Fields] OR "interleukin-2"[All Fields] OR "IL6"[All Fields] OR "IL-6"[All Fields] OR "interleukin-6"[All Fields] OR "IL8"[All Fields] OR "IL-8"[All Fields] OR "interleukin-8"[All Fields] OR "IL12"[All Fields] OR "IL-12"[All Fields] OR "interleukin-12"[All Fields] OR "IL17"[All Fields] OR "IL-17"[All Fields] OR "interleukin-17"[All Fields] OR "IL18"[All Fields] OR "IL-18"[All Fields] OR "interleukin-18"[All Fields] OR ("Lipids"[All Fields] OR "Lipid"[All Fields] OR "Lipid-lowering"[All Fields] OR "cholesterol"[All Fields] OR "Total cholesterol"[All Fields] OR "TC"[All Fields] OR "Low-density lipoprotein cholesterol"[All Fields] OR "LDL"[All Fields] OR "High-density lipoprotein cholesterol"[All Fields] OR "HDL"[All Fields] OR "Triglycerides"[All Fields] OR "Triglyceride"[All Fields] OR "TG"[All Fields] OR "Apolipoprotein"[All Fields] OR "Apolipoprotein-B"[All Fields] OR "apo b"[All Fields] OR "apo b"[All Fields] OR "Apolipoprotein-A1"[All Fields] OR "apo a1"[All Fields] OR "apo a1"[All Fields] OR "dyslipidemia"[All Fields] OR "dyslipidemias"[All Fields])) AND ("HIV"[All Fields] OR "Human Immunodeficiency Virus"[All Fields] OR "HIV"[All Fields] OR "HIV-positive"[All Fields] OR "AIDS"[All Fields] OR "Acquired Immunodeficiency Syndrome"[All Fields]) AND ("fish oil"[All Fields] OR "N-3"[All Fields] OR "ALA"[All Fields] OR ("eur policy anal"[Journal] OR "epa"[All Fields]) OR "DHA"[All Fields] OR "eicosapentaenoic acid"[All Fields] OR "docosahexaenoic acid"[All Fields] OR "omega 3"[All Fields] OR "omega 3"[All Fields] OR "PUFA"[All Fields] OR "Polyunsaturated fatty acids"[All Fields])

**WOS: 983 Results**

**WOS search:**

(TS= (("C-reactive protein" OR CRP OR ESR OR "Erythrocyte sedimentation rate" OR"tumor necrosis factor" OR TNF OR "tumour necrosis factor alpha" OR "TNF-alpha" OR inflammat* OR "inflammatory marker*" OR "inflammatory biomarker*" ORIL1 OR "IL-1" OR "interleukin-1" ORIL2 OR "IL-2" OR "interleukin-2" ORIL6 OR "IL-6" OR "interleukin-6" ORIL8 OR "IL-8" OR "interleukin-8" ORIL12 OR "IL-12" OR "interleukin-12" ORIL17 OR "IL-17" OR "interleukin-17" ORIL18 OR "IL-18" OR "interleukin-18")OR(Lipid* OR cholesterol OR "Total cholesterol" OR TC OR "Low-density lipoprotein cholesterol" OR LDL OR "High-density lipoprotein cholesterol" OR HDL OR Triglyceride* OR TG OR Apolipoprotein* OR "Apolipoprotein-B" OR "apo b" OR "Apolipoprotein-A1" OR "apo a1" OR dyslipidemia*))AND((HIV OR "Human Immunodeficiency Virus" OR "HIV-positive" OR AIDS OR "Acquired Immunodeficiency Syndrome"))AND(("fish oil" OR "N-3" OR ALA OR EPA OR DHA OR "eicosapentaenoic acid" OR "docosahexaenoic acid" OR "omega 3" OR PUFA OR "Polyunsaturated fatty acid*")))

**Scopus: 846 results**

**Scopus search:**

TITLE-ABS-KEY(((("C-reactive protein" OR CRP OR ESR OR "Erythrocyte sedimentation rate" OR"tumor necrosis factor" OR TNF OR "tumour necrosis factor alpha" OR "TNF-alpha" OR inflammat* OR "inflammatory marker*" OR "inflammatory biomarker*" ORIL1 OR "IL-1" OR "interleukin-1" ORIL2 OR "IL-2" OR "interleukin-2" ORIL6 OR "IL-6" OR "interleukin-6" ORIL8 OR "IL-8" OR "interleukin-8" ORIL12 OR "IL-12" OR "interleukin-12" ORIL17 OR "IL-17" OR "interleukin-17" ORIL18 OR "IL-18" OR "interleukin-18")OR(Lipid* OR cholesterol OR "Total cholesterol" OR TC OR "Low-density lipoprotein cholesterol" OR LDL OR "High-density lipoprotein cholesterol" OR HDL OR Triglyceride* OR TG OR Apolipoprotein* OR "Apolipoprotein-B" OR "apo b" OR "Apolipoprotein-A1" OR "apo a1" OR dyslipidemia*))AND((HIV OR "Human Immunodeficiency Virus" OR "HIV-positive" OR AIDS OR "Acquired Immunodeficiency Syndrome"))AND(("fish oil" OR "N-3" OR ALA OR EPA OR DHA OR "eicosapentaenoic acid" OR "docosahexaenoic acid" OR "omega 3" OR PUFA OR "Polyunsaturated fatty acid*"))))

**
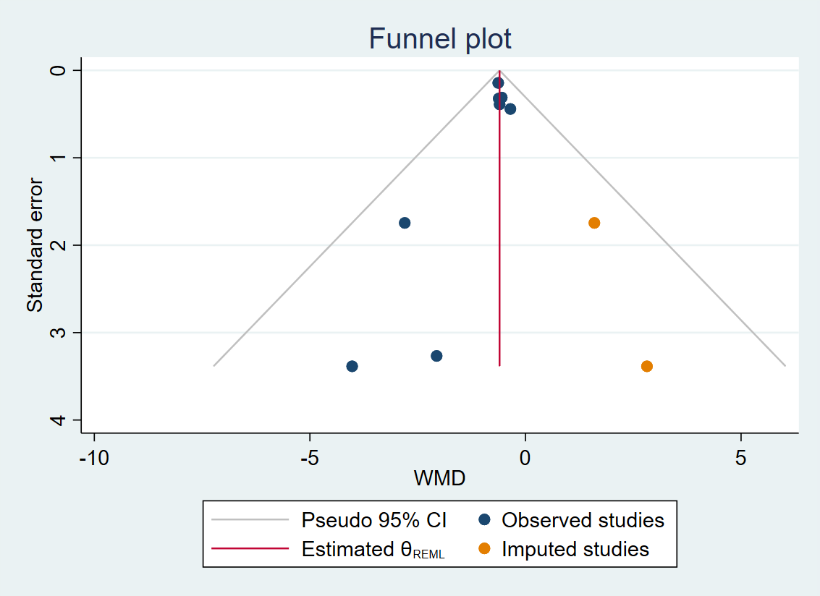
**

**Figure S1.** Trim and fill funnel plot for CRP


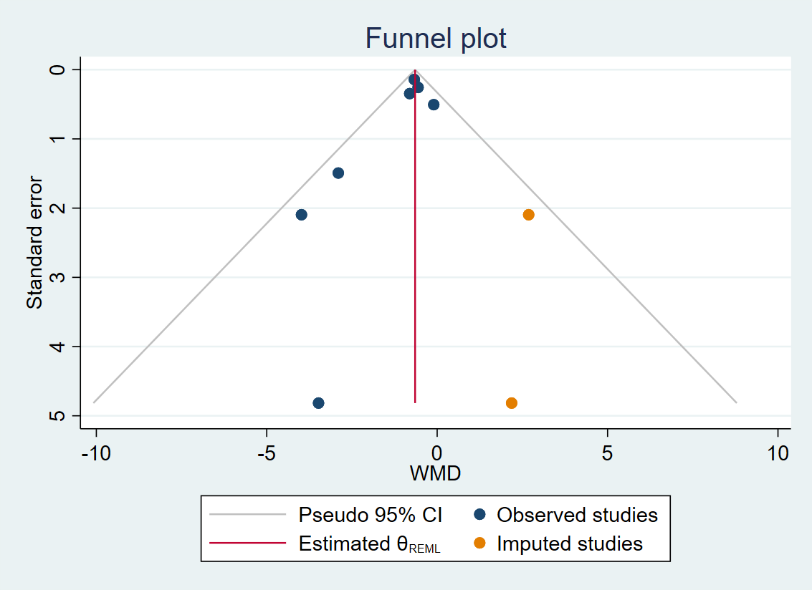


**Figure S2.** Trim and fill funnel plot for Il-6.


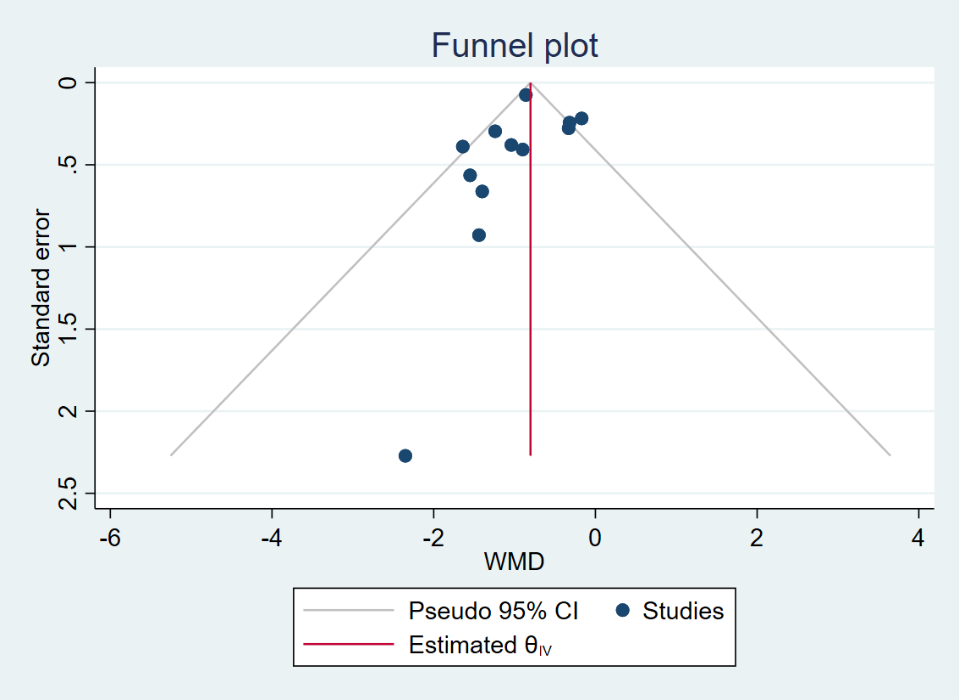


**Figure S3.** Trim and fill funnel plot for TG.

**
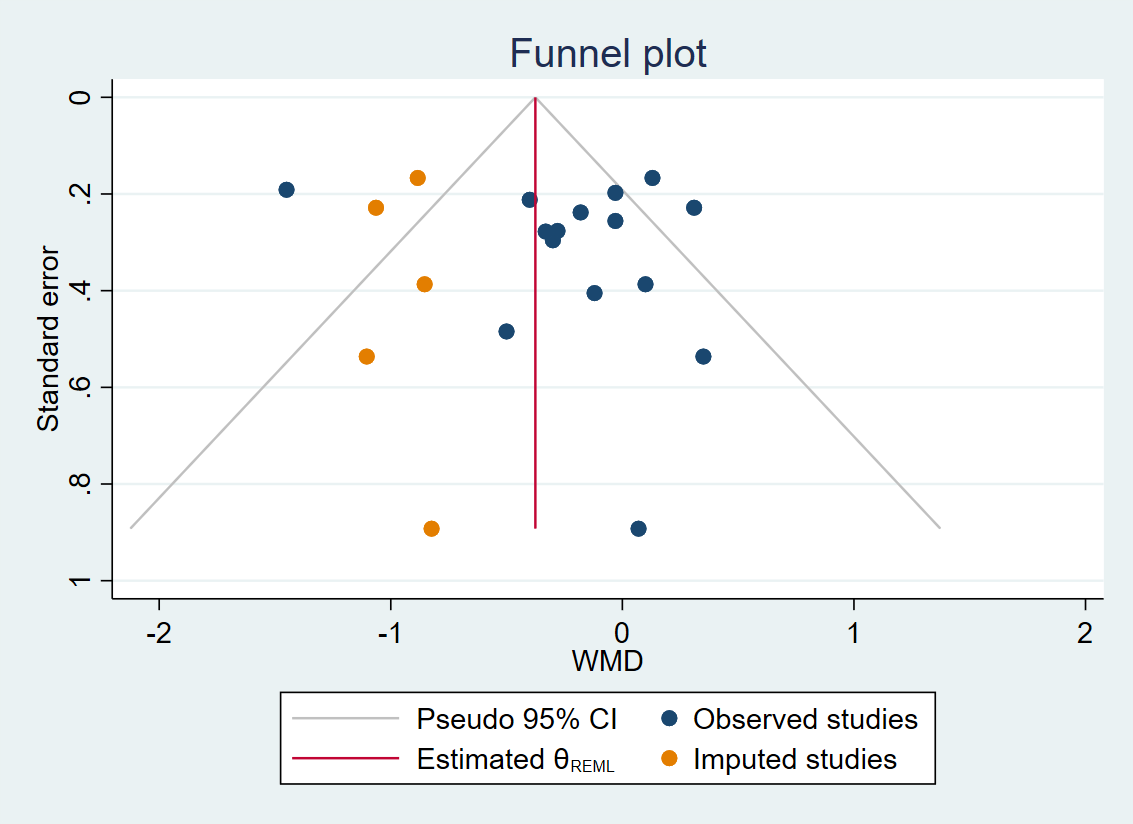
**

**Figure S4.** Trim and fill funnel plot for total cholesterol.

**
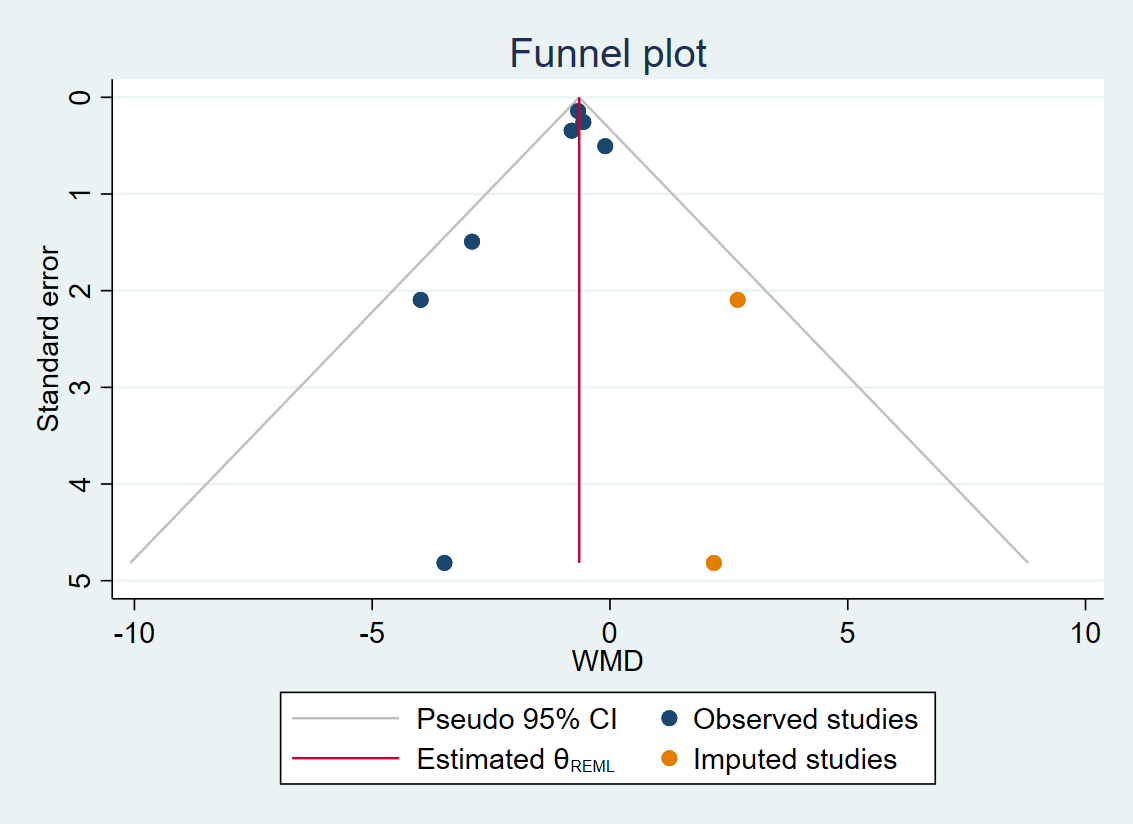
**

**Figure S5.** Trim and fill funnel plot for LDL.


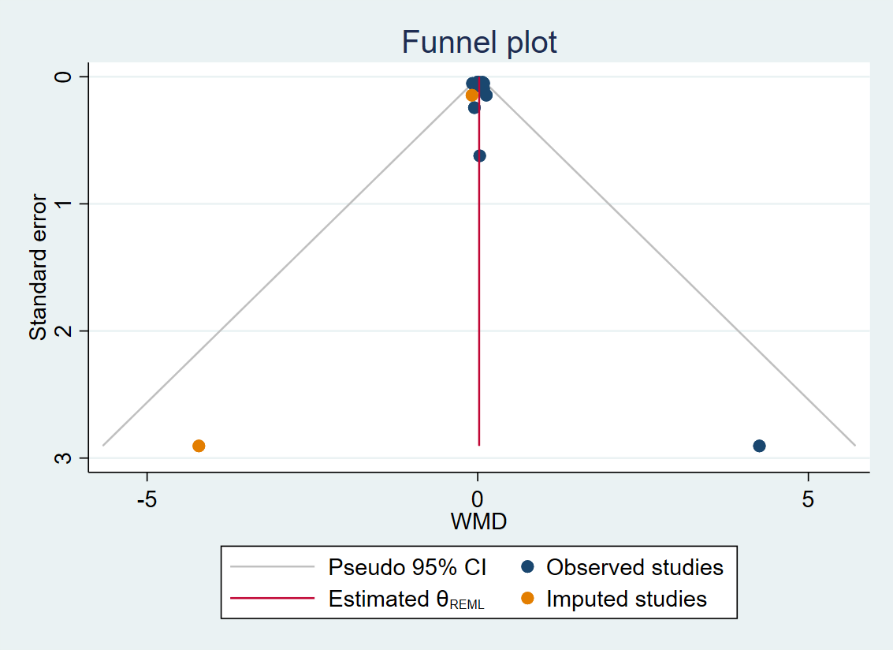


**Figure S5.** Trim and fill funnel plot for HDL.


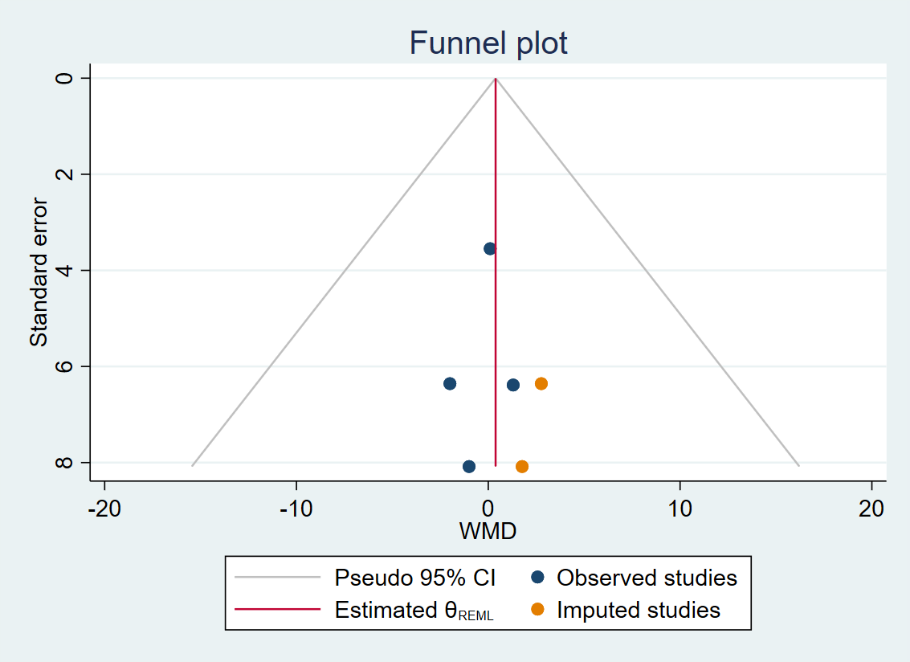


**Figure S6.** Trim and fill funnel plot for apolipoprotein A.

**
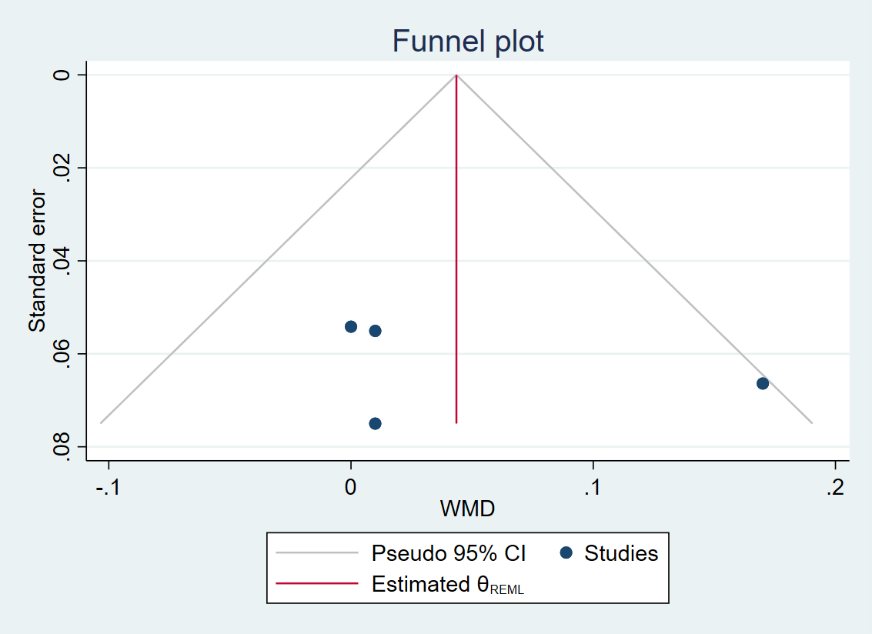
**

**Figure S7.** Trim and fill funnel plot for apolipoprotein B.
